# Implementation of Self-Initiated Event-Contingent Ecological Momentary Assessment for Environmental Exposures: Prospective Observational Study

**DOI:** 10.2196/96009

**Published:** 2026-08-18

**Authors:** Gregory Neely, Isabella Ström

**Affiliations:** 1 Department of Psychology, Umeå University, Mediagränd 14, Umeå, Västerbotten, 901 87, Sweden, 46 907869752; 2 Department of Behavioural Sciences and Learning, Linköping University, Linköping, Östergötland, Sweden

**Keywords:** ecological momentary assessment, smartphone app, compliance, reporting completeness, mobile health

## Abstract

**Background:**

Retrospective questionnaires are commonly used to assess exposure to unpleasant environmental stimuli, such as sounds and odors, but they are vulnerable to recall and salience biases. Ecological momentary assessment (EMA) is often proposed as a superior alternative because it captures experiences close in time to their occurrence. However, self-initiated, event-contingent EMA protocols require sustained participant engagement, and little is known about their feasibility, reporting completeness, and implementation challenges under naturalistic conditions.

**Objective:**

This study aims to evaluate a self-initiated smartphone-based EMA protocol for documenting unpleasant sound and odor exposures and to examine the implications of observed EMA use, the time between the first and last report, and reporting completeness for interpreting data obtained using the self-initiated EMA.

**Methods:**

Participants completed a 3-month study using a self-initiated, event-contingent smartphone-based EMA protocol alongside baseline and follow-up retrospective questionnaires. Individuals with and without self-reported environmental sensitivity to sounds or odors were offered access to a web-based EMA app for self-initiated reporting of exposure events. Reporting behavior measures included observed EMA use, the number of submitted reports, the time between the first and last submitted reports, reporting completeness (self-reported missed events), and barriers to app use. Analyses were primarily descriptive.

**Results:**

Of 104 participants (environmentally sensitive [ES], n=42; nonenvironmentally sensitive [NES], n=62), EMA reporting was limited, with 15 (36%) ES participants and 22 (35%) NES participants submitting at least one report (*χ*²_1_=0.00, *P*=.98). Report submission was concentrated early in the study period, and no participant submitted reports across the full 3-month observation period. Among EMA users, the mean reporting duration (time between the first and last submitted reports) was 19.9 (SD 17.6, range: 1‐51) days in the ES group and 7.9 (SD 9.5, range: 1‐32) days in the NES group. Missed exposure reports were common: 14 of 15 (93%) ES users and 14 of 22 (64%) NES users reported experiencing one or more exposure events that were not recorded using EMA. In addition, many nonusers reported unrecorded exposure events during the study period (ES: 11/27, 41%; NES: 5/40, 13%).

**Conclusions:**

Under naturalistic conditions, this 3-month self-initiated, event-contingent EMA protocol demonstrated limited observed use, declining report submissions over time, and substantial self-reported incompleteness. These findings arise from 1 specific implementation and should not be generalized to self-initiated EMA protocols using different sampling procedures, onboarding, participant support, reminders, incentives, or technologies. Nevertheless, they highlight the importance of reporting implementation characteristics when interpreting data from self-initiated, event-contingent EMA studies.

## Introduction

Accurate measurement of adverse environmental experiences is difficult because the outcomes of interest are not limited to the external exposure itself. Unpleasant sounds and odors are experienced in context, and their momentary impact depends not only on the physical stimulus but also on where the person is, what they are doing, and how the exposure is interpreted in daily life [1]. This contextual complexity is especially relevant in environmental intolerance research, where intolerance to odorous or pungent chemicals, sounds, buildings, and electromagnetic fields can co-occur rather than present as isolated complaints [2,3]. In people with self-reported electromagnetic hypersensitivity, for example, odor and noise intolerance are substantially elevated and strongly correlated, suggesting that adverse environmental experiences may cluster across sensory domains rather than map neatly onto a single exposure type [2].

Retrospective questionnaires remain important for population research because they are efficient and scalable, but they compress repeated events into summary judgments. Shiffman et al [4] argued that global retrospective reports are limited by recall bias and are poorly suited for examining how behavior and experience change across time and context. The same basic problem has been noted in environmental noise research, where annoyance is often measured as a long-term retrospective judgment, even though exposure and response vary across places and activities during the day [1]. For questions about when an unpleasant exposure occurred, how often it occurred, and under what circumstances it became bothersome enough to matter, retrospective summary measures therefore provide only a partial account.

Ecological momentary assessment (EMA) was developed to precisely address these limitations. EMA collects repeated reports of current behavior and experience in real time and in natural environments, thereby minimizing recall bias, maximizing ecological validity, and permitting the study of microprocesses that unfold in daily life [4]. As smartphone technology has matured, EMA has become increasingly feasible outside tightly controlled laboratory or clinical settings, and contemporary studies now routinely combine repeated self-report with mobile sensing, logging, or geolocation [5]. In environmental health research, these capabilities are especially attractive because they allow subjective reports to be linked to immediate context rather than to be inferred from delayed recall alone [1].

The migration from paper diaries to electronic and smartphone-based methods has also changed what reporting behavior problems are visible to researchers. Earlier diary work showed that paper records could appear complete while masking substantial backfilling or “hoarding,” whereas electronic systems incorporated compliance-enhancing features and made recording behavior more verifiable [6]. More recent methodological guidance has therefore treated EMA not only as a measurement strategy but also as an implementation problem involving software, devices, onboarding, training, prompting logic, data transmission, and participant support [7]. In other words, the validity of EMA data depends not only on what participants report but also on whether the protocol is actually used as intended in everyday life.

That implementation problem is now well-documented in the EMA literature. In a systematic review and meta-analysis of adult mobile EMA studies, Williams et al [8] found that compliance reporting was heterogeneous, often incomplete, and difficult to compare across studies; only 82 of 105 datasets reported compliance using a single metric, and pooled cohort compliance among eligible datasets was 81.9% with substantial heterogeneity. The same review noted that low compliance threatens ecological validity but also emphasized that acceptable completion rates do not guarantee accurate or representative data if missingness is patterned rather than random [8]. Wen et al [9] reached a similar conclusion in youth studies, arguing that better reporting of compliance is necessary not only to describe feasibility but also to determine whether missing data reflect random noise or systematic links to participant or situational characteristics. Collectively, these reviews show that “compliance” is not a simple descriptive statistic; it is part of the measurement process itself.

At the same time, the relation between protocol design and data completeness is not straightforward. Businelle et al [10] noted that prior meta-analytic findings on the effects of prompt frequency, study duration, and device type had been mixed, and then experimentally manipulated several common design features in a nationwide factorial study of smartphone EMA. Across a 28-day protocol, participants completed 83.8% of scheduled assessments, and the authors found no significant main effects of prompting 2 vs 4 times per day, using 15 vs 25 items, or using fixed vs random schedules on completion rates [10]. Williams et al [8] similarly concluded that total protocol burden has often been assumed to influence adherence, but that systematic evidence remains inconsistent. Methodological guidance has therefore shifted away from searching for a universally optimal EMA schedule and toward understanding how design choices interact with the target population, study context, and implementation procedures [7,10].

A second lesson from digital health research is that declining use over time should not be treated as a secondary nuisance. Eysenbach [11] argued that eHealth research requires a “science of attrition” because discontinuation and nonuse are common, consequential, and analytically meaningful rather than exceptional. This broader point resonates strongly with EMA. In a 9-week clinical trial, Tonkin et al [12] found that EMA compliance declined linearly across weeks and that the decline was steeper for some participant subgroups, including younger adults, full-time workers, and those who later dropped out of the study. For long-duration protocols, then, a final completion percentage can obscure an important dynamic process: engagement may erode gradually, selectively, and in ways that shape which experiences ultimately enter the dataset.

These issues become even more important when assessments are participant-initiated rather than prompt-driven. De Vries et al [5] distinguished time- or interval-contingent designs, in which participants are prompted at scheduled or random times, from event-contingent designs, in which participants are asked to respond when a predefined event occurs. In their review of smartphone-based EMA studies of well-being, time- or interval-contingent designs were by far the most common, whereas only a minority of studies used event-contingent or mixed designs [5]. A recent scoping review in hearing health care similarly describes EMA as encompassing time-based, interval-based, signal-based, event-contingent, and self-initiated approaches, underscoring that these sampling logics should not be treated as methodologically interchangeable [13], a point reinforced in an EMA methodology tutorial presented by Schinkel-Bielefeld et al [14].

For self-initiated event-contingent EMA, the data-generating process is especially demanding because the participant must do more than answer a prompt. The participant must notice that an eligible event has occurred, decide that it is worth reporting, interrupt ongoing activity, open the app, and complete the entry. Vercammen et al [15], who analyzed 8793 self-initiated EMAs from 2301 hearing aid wearers, showed the potential value of this approach: self-initiated smartphone reports captured detailed real-world experiences that could inform individualized care. At the same time, the same study highlighted its selectivity and vulnerability. Most users submitted only 1 to 4 entries; the data likely reflected a tech-savvy and motivated subgroup; and the authors explicitly noted that self-initiated EMA is prone to poor compliance when participants themselves must determine when an event of interest has taken place [15]. They also acknowledged that some entries may still have been completed retrospectively after the event, particularly when immediate phone use was impractical, which means that even “real-time” self-initiated systems can involve unobserved delays between experience and report [15].

Environmental sound and odor exposures are often episodic, context-dependent, and unpredictable, making them well-suited to event-contingent reporting. Real-time environmental EMA studies have shown that annoyance and stress vary across activities and contexts rather than simply reflecting average exposure levels [1,16]. Meanwhile, environmental intolerance research has primarily focused on the prevalence and overlap of different intolerance domains [2,3,17], and EMA methodology research has concentrated largely on prompted designs and compliance metrics [8-10]. As a result, relatively little is known about how people adopt, sustain, and use self-initiated smartphone-based EMA systems to document unpleasant environmental events over extended periods of everyday life.

The present study addressed that gap by examining the use of a smartphone-based, self-initiated, event-contingent EMA protocol designed to capture unpleasant sound and odor exposures during everyday life over a 3-month period. The primary aim was methodological rather than etiologic to understand how participants interacted with the reporting system and how that interaction shaped the completeness of the resulting EMA dataset. In addition to observed reporting behavior within the app, participants completed retrospective questionnaires at baseline and follow-up, which made it possible to compare recorded EMA entries with participants’ later accounts of exposures that were experienced but not entered into the app. This design allowed implementation to be examined as a multistage process involving initial EMA use, reporting behavior over time, and completeness of reporting when eligible events occurred.

More specifically, the study addressed 3 questions. First, to what extent did participants adopt a self-initiated smartphone EMA tool for documenting unpleasant sound and odor exposures? Second, to what extent did report submission continue across a 3-month naturalistic observation period? Third, to what extent did participants report experiencing eligible events that were not captured through EMA and what barriers contributed to those missing reports? By examining initial EMA use, reporting patterns over time, and reporting completeness, rather than relying on a single aggregate compliance percentage, the study aims to clarify the implications of implementation for the resulting EMA dataset.

## Methods

### Study Design

This study used a prospective observational design to examine the implementation of a self-initiated, event-contingent EMA protocol under naturalistic conditions. Participants completed baseline and follow-up questionnaires, and following the completion of the baseline assessment, they were offered access to a smartphone-based EMA app for documenting naturally occurring unpleasant sound and odor exposures over a 3-month period. Rather than estimating the prevalence or frequency of environmental exposures, the study focused on methodological outcomes describing participants’ engagement with the EMA protocol, including initial EMA use, reporting patterns over time, reporting completeness, and self-reported barriers to EMA use.

### Participants and Recruitment

Participants were recruited from the Västerbotten Environmental Health Study (VEHS), a population-based cohort originally comprising 8600 randomly selected adults from Västerbotten County [3]. The cohort had completed 3 questionnaire waves over approximately 6 years. Individuals who completed all 3 waves (n=1837) and had access to a smartphone or other mobile devices capable of running the web-based EMA app were eligible for participation. No formal a priori sample size calculation was conducted because the study had a descriptive methodological focus and sought to evaluate the implementation of a self-initiated EMA protocol within an existing cohort.

Eligible participants received a postal invitation containing written information describing the purpose of the study, study procedures, voluntary participation, and an informed consent form together with a prepaid return envelope. Individuals who did not initially respond received a reminder letter. Of the 1837 eligible individuals, 341 could not be contacted because they had moved or died, leaving 1496 individuals successfully contacted.

Participants were classified into environmentally sensitive (ES) group and nonenvironmentally sensitive (NES) groups based on responses to the baseline questionnaire administered as part of the present study. Individuals reporting sensitivity to sounds or odors were classified as ES, whereas those reporting no such sensitivities were classified as NES. Classification was based solely on baseline responses collected for the present study and was independent of responses provided during previous VEHS survey waves. A total of 143 individuals consented to participate (n=55 ES and n=88 NES).

### EMA System and Measures

#### EMA Reporting Tool

Data were collected using a custom-developed EMA app designed for real-time capture of environmental exposures and associated symptoms in daily life [18]. The app was compatible with standard smartphones and communicated with a secure web-based server for storage and subsequent retrieval of submitted reports. Participants were instructed to save the application as a shortcut on the home screen of their devices to facilitate rapid access.

The app was intentionally designed to minimize reporting burden during everyday activities. The completion of an initial report typically required approximately 45 to 60 seconds and consisted of 3 sequential screens. Participants first recorded event-specific information, including the type of exposure, time of occurrence, and perceived discomfort, using a 10-point numerical rating scale. The second screen captured symptom data using a predefined, hierarchical symptom list based on the Environmental Hypersensitivity Symptom Inventory, allowing users to select symptom categories and specific symptoms [2]. The final screen collected contextual metadata (eg, location) and allowed optional free-text input to support later recall. The app allowed participants to self-initiate reports whenever they experienced a sound or odor that caused discomfort in their daily environment. The application supported offline data entry, with automatic synchronization to the server once an internet connection was available. Participants could subsequently access a secure web interface to review and complement their reports, including providing follow-up information on symptom duration using predefined response categories.

#### Retrospective Questionnaires

Participants completed questionnaires immediately before the EMA period (baseline) and following the completion of the 3-month observation period (follow-up). The questionnaires assessed demographic characteristics, self-reported environmental intolerance, the perceived frequency of exposure to unpleasant sounds and odors, and associated symptoms.

The follow-up questionnaire additionally contained structured questions regarding participants’ experiences using the EMA app, including whether they had chosen not to use the application, whether they had experienced exposure events that were not reported, and the perceived reasons for nonuse or missed reporting.

#### Procedure

Following written informed consent and completion of the baseline questionnaire, participants received written instructions describing installation and the use of the EMA app together with individual login credentials. Participants were instructed to submit an EMA report whenever they experienced an unpleasant sound or odor in their everyday environment. They were asked to report events as soon as practically possible after the exposure occurred but they were specifically instructed not to interrupt activities requiring attention, such as driving, in order to complete a report. If immediate reporting was not possible, participants were encouraged to submit the report as soon as circumstances permitted.

The EMA protocol was entirely self-initiated and event contingent. Participants were free to determine whether an exposure warranted reporting, and no minimum number of reports was required. No reminder notifications, scheduled prompts, or compliance-monitoring procedures were used during the 3-month observation period. Participants were informed that their participation was voluntary throughout the study and that they could discontinue reporting at any time without explanation.

Participants could access a secure web interface throughout the study period to review previously submitted reports and supplement them with additional information, if desired. Technical support was available by telephone and email during the study, although the research team did not undertake any proactive contact regarding reporting frequency or participant engagement.

After 3 months, participants completed the follow-up questionnaire, which included items concerning their experiences using the EMA app, perceived barriers to reporting, and reasons for nonuse or incomplete reporting. Participants received no financial compensation or other incentives for participation or for submitting EMA reports.

#### EMA Reporting Behavior Outcomes

Because the objective of the study was methodological rather than epidemiological, the primary outcomes described participants’ engagement with the EMA protocol rather than the characteristics of environmental exposures themselves.

Reporting behavior outcomes comprised 3 complementary dimensions of engagement. Initial EMA use was defined as submitting at least 1 EMA report during the study period. Reporting over time was assessed as the number of days between each participant’s first and last submitted EMA report. Reporting completeness was assessed retrospectively using follow-up questionnaire items asking participants whether they had experienced unpleasant sound or odor exposures that they had not reported using the EMA app.

Additional descriptive outcomes included the total number of submitted EMA reports per participant and self-reported reasons for nonuse or incomplete reporting. Reported barriers were categorized as technical (eg, app or device problems) or subjective (eg, forgetting, lack of motivation, or perceived reporting burden).

### Data Analysis

One participant in the NES group was excluded because they reported that they had never received the app instructions and therefore had never downloaded the EMA app.

Because the primary objective was to characterize the implementation of a self-initiated EMA protocol rather than estimate the population frequency of environmental exposures, analyses primarily focused on the descriptive characterization of participant engagement. Initial EMA use was summarized as the proportion of participants submitting at least 1 EMA report. Reporting over time was described using reporting duration and total reporting volume among participants who submitted at least 1 EMA report. Differences in initial EMA use between the ES and NES groups were examined using chi-square tests. In addition, an exploratory logistic regression analysis was conducted to examine whether age, gender, and environmental sensitivity status were associated with initial EMA use and to obtain descriptive estimates with corresponding CIs. Reporting duration and reporting volume were summarized descriptively because of the limited number of EMA users who submitted EMA reports and the nonrandom nature of app use.

Patterns of nonreporting, attrition, and retrospectively reported missed exposure events were interpreted as implementation outcomes rather than being treated solely as missing observations requiring statistical adjustment. Consequently, no attempt was made to estimate exposure frequency after accounting for missing EMA reports. Instead, analyses were intended to describe how participant engagement influenced the observations captured by the self-initiated EMA protocol.

### Ethical Considerations

The study was conducted in accordance with the Declaration of Helsinki. All participants received written information describing the study and provided written informed consent prior to participation. Participant identities were stored separately from study data, and all electronic data were maintained on secure servers with restricted access. Participants received no financial compensation for participation. The study was approved by the Regional Ethics Review Board in Umeå, Sweden (DNR 2018-111-31).

## Results

### Participant Characteristics

**Figure 1.**
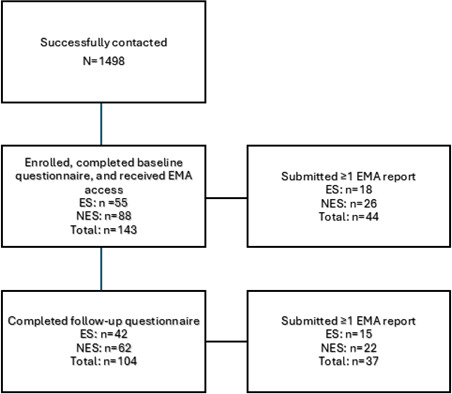
Participant flow through the study stratified by environmentally sensitive (ES) and nonenvironmentally sensitive (NES) groups. EMA: ecological momentary assessment.

Of the initial 143 participants, 104 completed both the baseline and follow-up questionnaires and constituted the main study sample (Figure 1).

Sample characteristics of those who completed the entire protocol are presented in Table 1. The relatively high mean age of participants (60.3, SD 10.9 y) reflected the demographic composition of the original VEHS cohort.

**Table 1.** Sample characteristics by group, gender, and age^a^.

Group	Female		Male		Total	
	Participants, n	Age (y), mean (SD)	Participants, n	Age (y), mean (SD)	Participants, n	Age (y), mean (SD)
ES^b^	31	54.3 (10.2)	11	65.6 (12.8)	42	57.3 (11.9)
NES^c^	22	58.4 (8.5)	40	64.7 (9.9)	62	62.5 (9.8)

a Values are mean (SD) unless otherwise indicated.

b ES: environmentally sensitive.

c NES: nonenvironmentally sensitive.

### Observed EMA Use

The use of the self-initiated EMA protocol was limited. Of the 143 participants who enrolled and received access to the EMA app, 44 (31%) submitted at least one EMA report during the study period (Figure 1). Seven of these participants did not complete the follow-up questionnaire. Consequently, analyses requiring follow-up data were restricted to participants who both completed the follow-up questionnaire and submitted at least one EMA report. Baseline characteristics of participants who completed and did not complete the follow-up questionnaire are presented in Multimedia Appendix 1. Participants who completed the follow-up questionnaire did not differ significantly from noncompleters with respect to age (Welch *t*_55.4_=−0.98, *P*=.33), gender (*χ*²_1_=0.02, *P*=.89), or environmental sensitivity status (*χ*²_1_=0.60, *P*=.44).

Among the 104 participants who completed both baseline and follow-up questionnaires, 37 (36%) submitted at least one EMA report. Fifteen (36%) participants in the ES group and 22 (35%) participants in the NES group submitted at least one EMA report during the 3-month observation period (Table 2). EMA reporting did not differ significantly between groups (*χ*²_1_=0.00, *P*=.98). Of the participants who completed follow-up but did not submit an EMA report, 28 (44%) reported that they had not experienced any qualifying unpleasant sound or odor exposures during the study period. The remaining participants reported experiencing one or more qualifying exposures that were not recorded using the EMA app (Table 2).

**Table 2.** EMA^a^ reporting and self-reported, unrecorded exposure events among participants who completed both baseline and follow-up questionnaires (N=104).

EMA usage and exposure experience	ES^b^, n	NES^c^, n
Used the EMA app (≥1 report)	15	22
Experienced ≥1 exposure not recorded	14	14
Did not use the EMA app	27	40
Reported no qualifying exposure during study	4	24
Reported ≥1 qualifying exposure not recorded	11	5

a EMA: ecological momentary assessment.

b ES: environmentally sensitive.

c NES: nonenvironmentally sensitive.

As an exploratory analysis, a logistic regression was conducted to examine whether age, gender, and environmental sensitivity status were associated with initial EMA use (submission of at least one EMA report). Given the small number of participants who submitted EMA reports (n=37), the analysis was not intended to provide definitive estimates of association. The model explained little of the variance in initial EMA use (Nagelkerke *R*²=0.04), and no predictor was statistically significant (age: odds ratio [OR] 0.97, 95% CI 0.93‐1.01; *P*=.15; environmental sensitivity: OR 0.80, 95% CI 0.32‐1.98; *P*=.62; gender: OR 1.20, 95% CI 0.47‐3.06; *P*=.70). These estimates should be interpreted cautiously because of the limited sample size.

### Reporting Over Time

Among participants who submitted at least one EMA report, the mean reporting duration, defined as the number of days between the first and last submitted EMA reports, averaged 19.9 (SD 17.6, range: 1‐51) days in the ES group and 7.9 (SD 9.5, range: 1‐32) days in the NES group. No participant submitted reports for the entire 3-month observation period.

**Figure 2.**
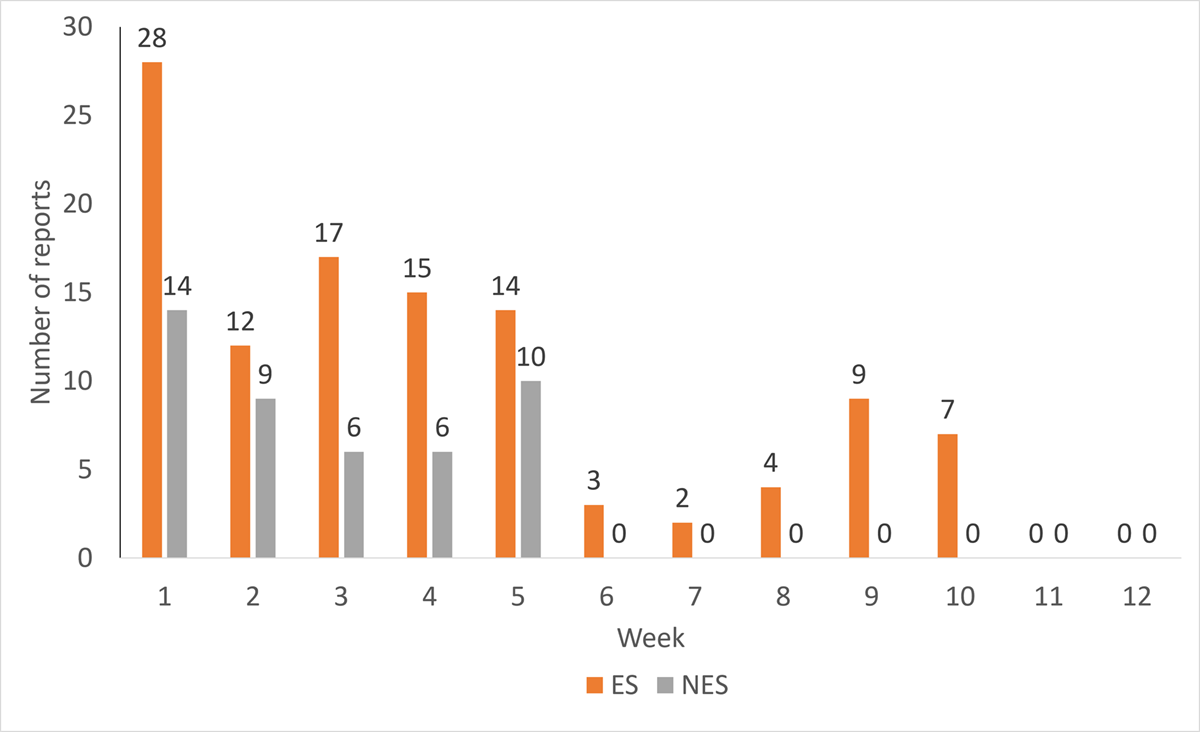
Weekly number of submitted ecological momentary assessment (EMA) reports over the 12-week reporting period, stratified by environmentally sensitive (ES) and nonenvironmentally sensitive (NES) groups. Counts represent the number of submitted reports rather than the frequency of environmental exposures.

Figure 2 illustrates the weekly distribution of submitted EMA reports. Reporting volume decreased markedly after the initial weeks in both groups. Among NES participants, few reports were submitted after week 6, whereas ES participants continued to submit occasional reports in later weeks, although at substantially reduced frequencies.

Participants in the ES group tended to submit more reports per person (median 4, IQR 2‐9) than participants in the NES group (median 1, IQR 1‐3). However, the number of submitted reports declined over the observation period, and no participant continued reporting throughout the entire 3-month study.

### Reporting Completeness

Retrospective responses collected at follow-up indicated that incomplete reporting was common among both EMA users and nonusers (Table 2). Among participants who had used the EMA app, 14 of 15 (93%) ES participants and 14 of 22 (64%) NES participants reported experiencing one or more unpleasant sound or odor exposures that had not been documented in the app.

Incomplete reporting was also evident among participants who had never adopted the EMA system. Among nonusers, 11 of 27 (41%) ES participants and 5 of 40 (13%) NES participants indicated that they had experienced at least 1 unpleasant exposure during the study period, despite not submitting any EMA reports. In contrast, 4 (15%) ES participants and 24 (60%) NES participants reported that they had not experienced any unpleasant sound or odor exposure during the observation period.

Participants who acknowledged having missed one or more reports were asked to indicate why the exposure had not been documented (Table 3). Across both environmental sensitivity groups and irrespective of EMA use, forgetting to report the event was the most frequently endorsed explanation. Fewer participants reported technical difficulties, lack of access to their phones at the time of exposure, or loss of motivation.

**Table 3.** Self-reported reasons for missed ecological momentary assessment (EMA) reports among participants reporting one or more unrecorded exposure events by environmental sensitivity group and EMA use status^a^.

Reason for missed report	ES^b^		NES^c^	
	Users (n=14)	Nonusers (n=11)	Users (n=14)	Nonusers (n=5)
Did not have phone available	1	4	1	0
App or phone not working properly	2	1	1	0
Forgot to report	10	5	9	3
Lost motivation	4	5	2	1

a Counts reflect endorsed explanations for missed reporting. Participants could select more than one response option; therefore, the total number of responses exceeds the number of respondents and percentages are not shown.

b ES: environmentally sensitive.

c NES: nonenvironmentally sensitive.

### Barriers to Initial Adoption

Participants who did not use the EMA app despite experiencing at least 1 exposure to unpleasant sound or odor during the study period were asked to indicate why they had not begun using the EMA app (Table 4). Multiple responses were permitted.

Technical barriers were reported more frequently by participants in the ES group than by participants in the NES group. This included difficulty locating the app on the phone, log-in problems, and uncertainty regarding the app use. Participants in both groups reported similar frequencies of discomfort with the reporting method and unfamiliarity with using smartphones for this type of documentation.

Participants could also provide open-ended comments describing other reasons for not using the app. These responses most commonly described technical difficulties, contextual constraints, and motivational factors. Comments from participants in the ES group reflected a broader range of barriers to EMA use, including technical problems, difficulties incorporating the app into everyday smartphone use, forgetfulness, and perceptions that the application was not relevant in certain situations. Participants in the NES group more frequently described practical barriers, such as lack of time or competing daily activities.

**Table 4.** Self-reported barriers to initial adoption of the ecological momentary assessment (EMA) app^a^.

Reason for not using the app	ES^b^, n	NES^c^, n
I could not find the app on my phone	4	2
I could not log in to the app	5	1
I did not understand how to use the app	2	0
I am not used to using my phone in this manner	2	4
This felt like a difficult or uncomfortable way to document experiences	6	7
Other	13	5

a Counts reflect endorsed response categories. Participants could select more than 1 reason; therefore, totals exceed the number of respondents and percentages are not reported.

b ES: environmentally sensitive.

c NES: nonenvironmentally sensitive.

## Discussion

### Principal Findings

This study evaluated the implementation of a 3-month self-initiated event-contingent EMA protocol under naturalistic conditions. The observed EMA use was limited, submitted reports declined over time, and many participants acknowledged unreported exposure events during follow-ups. These findings suggest that the resulting dataset reflects both environmental exposures and participant engagement with the reporting process.

Our findings suggest that implementation characteristics warrant careful consideration when evaluating self-initiated EMA protocols. Much of the EMA literature has focused on prompted designs, in which compliance can be quantified directly as the proportion of scheduled assessments completed [4,9,19]. In contrast, self-initiated event-contingent EMA requires participants to recognize reportable events, decide that reporting is warranted, and initiate documentation without external prompting. Vercammen et al [15] similarly demonstrated the feasibility of self-initiated EMA for capturing hearing-aid experiences in daily life and also reported a relatively modest reporting frequency. The current study adds a further perspective by suggesting that initial EMA use, reporting patterns, and reporting completeness may each influence the observations ultimately available for analysis under naturalistic conditions.

Retrospective questionnaires remain susceptible to reconstructive memory, recall bias, and the disproportionate influence of salient experiences on later reports [20-22]. EMA was developed specifically to reduce these limitations by collecting information closer to the time of the experience [4]. The present findings indicate that reducing recall-related error does not eliminate the need to consider how observations are generated. In self-initiated event-contingent protocols, whether participants begin using the system, how they report over time, and whether eligible events are reported all influence which experiences ultimately become recorded observations.

One of the clearest patterns observed was the decline in submitted reports over the 3-month observation period. Even among participants who submitted reports later in the study, many reported at follow-up that they had experienced exposure events that were never documented. Because the protocol was event-contingent, this pattern cannot be attributed to any single mechanism. It may reflect changes in the frequency of qualifying events, participants’ thresholds for deciding that an event warranted reporting, missed reports, or reduced engagement with the protocol. The methodological importance of incomplete reporting lies not only in the loss of observations but also in the possibility that unreported events could differ systematically from those that are recorded. Because self-initiated event-contingent EMA provides no direct record of eligible events that were never reported, missing observations may reflect characteristics of participants, situations, or the events themselves rather than random omission. This possibility has important implications for statistical analysis. Ma et al [23] have argued that this presents a distinct methodological challenge for self-initiated EMA because conventional approaches to handling missing data are often difficult to apply when the occurrence of unreported events is itself unobserved. Although this study cannot determine whether unreported events differed systematically from reported events, the substantial proportion of retrospectively acknowledged missed reports underscores the importance of considering reporting completeness when interpreting self-initiated EMA datasets.

This pattern is consistent with a broader literature describing declining engagement in digital health interventions over time. Eysenbach [11] “Law of Attrition” proposed that decreasing participation is an inherent characteristic of many eHealth interventions rather than simply an unfortunate methodological complication. More recent research has similarly shown that engagement with EMA protocols is influenced by study duration, reporting burden, protocol complexity, and characteristics of the study population [9,10,12]. The present study is consistent with the broader literature in suggesting that, in self-initiated event-contingent EMA, participant engagement involves not only remaining active over time but also consistently translating experienced events into recorded observations.

The barriers reported by participants suggest several plausible contributors to incomplete reporting rather than pointing to a single explanation. Forgetting to report was the most frequently endorsed explanation for missed events, while technical difficulties, contextual constraints, and motivational factors were reported less frequently but nevertheless contributed to incomplete documentation. Similar challenges have been identified in methodological discussions of EMA implementation, where participant burden, integration of reporting into everyday routines, and balancing ecological validity against reporting demands are recognized as important considerations in the design of self-initiated protocols [14]. Consistent with this perspective, participants in the present study were explicitly instructed not to interrupt activities requiring attention in order to complete an EMA report, and the protocol deliberately incorporated no reminder notifications, proactive monitoring, or engagement support to approximate naturalistic use over an extended observation period. Under these conditions, incomplete reporting should be viewed as an expected feature of this implementation rather than simply interpreted as evidence of poor study conduct. Different implementation choices might reasonably produce different patterns of participant engagement.

### Methodological Implications

The limited initial EMA use, declining report submissions over time, and high frequency of retrospectively acknowledged missed events observed here have several implications for self-initiated EMA studies. Initial EMA use, reporting patterns over time, and reporting completeness should be regarded not only as descriptive characteristics of participant engagement but also as information that helps readers evaluate how the resulting observations were generated, and consequently, what they may represent. In self-initiated event-contingent EMA, unreported events are typically unobservable, making it difficult to determine whether incomplete reporting reflects random omissions or more systematic patterns of nonresponse. As Ma et al [23] have argued, this feature distinguishes self-initiated EMA from many prompted designs and complicates interpretation of missing data. Although this study cannot determine whether missed reports occurred systematically, documenting these aspects of participant engagement provides important context for interpreting the completeness and potential representativeness of the resulting dataset. Reporting these measures alongside conventional descriptions of the study sample and assessment protocol would therefore improve transparency and facilitate comparisons across studies using different EMA designs.

Participant engagement should be considered during the design of self-initiated event-contingent EMA studies rather than only when evaluating study performance after data collection. Decisions regarding study duration, reporting burden, user interface design, participant training, reminder strategies, and technical support have all been identified as factors that may influence engagement with EMA protocols [9,10,12,24]. Much of this evidence, however, comes from prompted EMA designs, where scheduled assessments support engagement and missed observations are directly observable. In contrast, self-initiated event-contingent EMA requires participants to recognize reportable events, decide whether reporting is appropriate, and integrate reporting into everyday life. Design principles developed for prompted protocols may therefore not translate directly to self-initiated approaches.

Additionally, participant burden should be viewed more broadly than questionnaire length or reporting frequency alone. Although the EMA reports required less than 1 minute to complete, participants described difficulties in integrating reporting into their daily routines. Forgetting to report was the most frequently endorsed explanation for incomplete documentation, while contextual factors such as not having the phone readily available also contributed. Methodological guidance for self-initiated EMA has similarly emphasized that participant burden reflects not only the duration of individual assessments but also how easily reporting can be incorporated into everyday activities and routines [14]. Consistent with this perspective, the present findings suggest that reporting process burden reflects the cumulative demands imposed by repeated decision-making and behavioral interruption rather than simply the time required to complete an individual assessment. Future studies may therefore benefit from considering implementation as a behavioral process in which convenience, habit formation, and contextual fit are likely to influence reporting along with aspects such as interface usability.

The relatively older age of the study population should also be considered when interpreting the findings. Although age was not significantly associated with EMA use in the present study, the sample was older than many cohorts typically included in digital health research. Smartphone ownership among older adults has increased substantially during the past decade [25]. However, familiarity with integrating mobile technologies into everyday self-monitoring remains highly variable [26]. Methodological guidance for EMA has also noted that age-related differences in digital familiarity and technology use should be considered when designing self-initiated protocols [14]. Therefore, the present findings do not indicate that age itself explains the observed reporting patterns, but they suggest that characteristics of the intended user population should be considered when assessing the transferability of self-initiated EMA protocols to other settings.

Participants’ use of EMA deserves greater attention in methodological reporting standards for self-initiated event-contingent studies. Reporting only the number of completed observations provides an incomplete picture of how those observations were generated and what experiences may ultimately be represented in the resulting dataset. Reporting these implementation measures alongside conventional compliance metrics would improve transparency and facilitate comparisons across studies.

### Strengths, Limitations, and Future Directions

This study has several strengths. It evaluated a self-initiated, event-contingent EMA protocol under naturalistic conditions over a relatively long observation period, allowing implementation to be examined in a context that closely resembled intended real-world use. By combining prospective EMA with retrospective follow-up questionnaires, the study was also able to compare recorded observations with participants’ own reports of events that had not been documented. This provided information about reporting completeness that is generally unavailable in studies relying exclusively on EMA data. In addition, inclusion of participants with and without self-reported environmental sensitivity allowed reporting behavior to be examined across groups expected to differ in the frequency and personal relevance of unpleasant environmental exposures.

Several limitations should also be considered when interpreting the findings. First, the sample size was modest, particularly among participants who actively used the EMA app, limiting opportunities for subgroup analyses and formal modeling of factors associated with implementation. Second, the study population was relatively older, reflecting the demographic characteristics of the VEHS cohort. Although this provided an opportunity to examine EMA implementation in a population that is often underrepresented in digital health research, the findings may not generalize directly to younger populations with different patterns of smartphone use.

Third, reporting completeness was assessed retrospectively. Participants’ reports of missed exposure events are themselves subject to memory limitations and therefore cannot provide an exact estimate of the number of undocumented events. Consequently, the study cannot determine the true magnitude of incomplete reporting. Rather, the retrospective responses demonstrate that participants recognized the existence of unreported events, indicating that the EMA dataset should not be interpreted as a complete record of all eligible exposures.

Additionally, the study is subject to potential selection at multiple stages. Individuals first self-selected by consenting to participate, a proportion did not complete the follow-up questionnaire, and only a subset subsequently adopted the EMA protocol. As a result, the observations analyzed in this study were generated from participants who successfully progressed through several stages of participation and may not be representative of the broader eligible cohort. The findings should therefore be generalized with appropriate caution. Finally, exploratory analyses examining associations with initial EMA use should be interpreted cautiously because the limited number of participants who submitted EMA reports resulted in imprecise estimates and limited statistical power.

The study evaluated one specific version of a self-initiated event-contingent EMA protocol: a 3-month assessment period with written onboarding, no prompts or reminder notifications, no minimum reporting requirement, no financial incentives, and no proactive monitoring or engagement support. Different app designs, sampling and reporting procedures, or participant support strategies might have produced different patterns of engagement and reporting. Accordingly, the findings should be interpreted as applying to this implementation rather than being assumed to characterize self-initiated EMA protocols more generally.

A relevant next step is to determine whether the implementation patterns observed here are replicated across different populations, health conditions, research settings, and EMA designs. Comparisons between self-initiated, event-contingent protocols and prompted protocols may be particularly informative because these approaches place different cognitive and behavioral demands on participants and are therefore likely to differ in how real-world experiences are translated into recorded observations.

### Conclusions

Self-initiated event-contingent EMA provides opportunities to document environmental exposures in everyday life that cannot readily be captured using retrospective questionnaires alone. In this study, EMA data were shaped not only by exposure events but also by participants’ engagement with the reporting process. Based on the present findings, researchers using self-initiated event-contingent EMA should consider reporting implementation characteristics alongside study outcomes to improve transparency and interpretation.

## Supplementary material

10.2196/96009 Multimedia Appendix 1 Baseline characteristics of participants who completed and did not complete the follow-up questionnaire.

## Abbreviations

EMA: ecological momentary assessment
ES: environmentally sensitive
NES: nonenvironmentally sensitive
OR: odds ratio
VEHS: Västerbotten Environmental Health Study
