# Supplementary material for: Implementation of Self-Initiated Event-Contingent Ecological Momentary Assessment for Environmental Exposures: Prospective Observational Study
Source: JMIR Form Res. 2026 Aug 18;10:e96009. doi: 10.2196/96009 (PMC13484943; doi:10.2196/96009)
Supplement: Supplementary file 1 [file formative-v10-e96009-s001.docx]

**Supplementary Table S1 Baseline characteristics of participants who completed and did not complete the follow-up questionnaire.**

| **Characteristic** | **No follow-up (n = 39)** | **Follow-up (n = 104)** | **p-value** |
| --- | --- | --- | --- |
| Age, years, mean (SD) | 57.6 (14.4) | 60.0 (10.9) | .33¹ |
| Female, n (%) | 20 (51.3) | 52 (50.0) | .89² |
| Environmentally sensitive (ES), n (%) | 13 (33.3) | 42 (40.4) | .44² |

*¹ Welch's independent-samples t-test.
² Chi-squared test.*
